# Prospects of Risk-Sharing Agreements for Innovative Therapies in a Context of Deficit Spending in Bulgaria

**DOI:** 10.3389/fpubh.2015.00064

**Published:** 2015-04-22

**Authors:** Georgi Iskrov, Rumen Stefanov

**Affiliations:** ^1^Department of Social Medicine and Public Health, Faculty of Public Health, Medical University of Plovdiv, Plovdiv, Bulgaria; ^2^Institute for Rare Diseases, Medical University of Plovdiv, Plovdiv, Bulgaria

**Keywords:** Bulgaria, deficit spending, drug budget, innovative therapies, uncertainty, evidence, risk-sharing, health technology assessment

## Funding of Innovative Therapies in Bulgaria

Innovative therapies are usually defined as newly introduced or modified health technologies with unproven effect or side effect undertaken in the best interest of the patient. These therapies could be situated at any point of the continuum: from genuine innovation with no precedent, to relative innovation representing a small variation from standard therapy, or using a conventional treatment in a different context (1). While the conception of innovative health technologies is not limited by therapeutic form (drugs, devices, procedures) or disease indication, innovative therapies are generally associated with expensive original drugs (2). Inclusion and provision of these therapies tend to be one of the most resource-consuming tasks for national health systems and payers.

The above mentioned perceptions are well illustrated by the National Health Insurance Fund (NHIF) in Bulgaria and its funding activities. NHIF is an independent public entity that was established to carry out the mandatory health insurance in the country. Progress in medical science and introduction of innovative therapies, together with aging population and increased prevalence of chronic non-communicable diseases have put NIHF into a permanent situation of budget deficit. Overspending has led to concerns about the overall sustainability of NHIF and the present health insurance model in Bulgaria. Moreover, NHIF is currently lacking effective mechanisms to address this growing financial risk.

**Figure 1:**
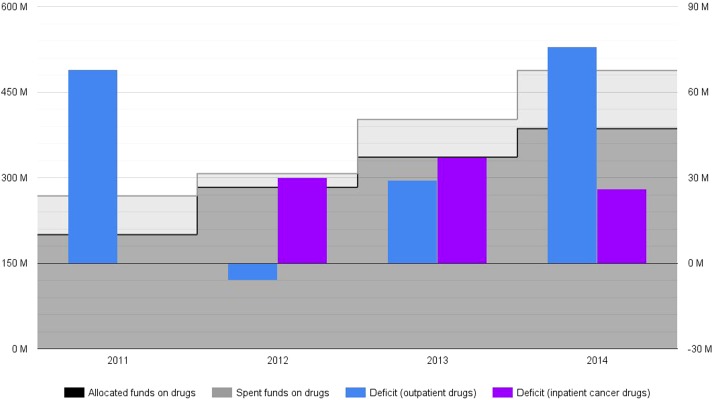
National Health Insurance Fund drug budget deficit for 2011–2014*^,^ ** (actual, non-discounted costs, reported in million EUR). *No actual spending on inpatient cancer drugs is reported for 2011, as these medicines were made part of (and subsequently paid by) NHIF basket in 2012. **The amount of actual spending for 2014 is based on forecasts.

National Health Insurance Fund budget is annually set and approved through a legal act by the National Assembly of Bulgaria. Its funds that are intended to cover drug therapies are distributed between two cost items as defined by the relevant legislation – costs for outpatient drugs and costs for inpatient cancer drugs. The first category mainly includes outpatient medicinal therapies, although a limited part of these funds are earmarked to medical devices and medical foods. NHIF total drug expenditure steadily rose between 2011 and 2014 (Figure 1). These total costs were 268 million EUR in 2011 and were expected to reach up to 488 million EUR in 2014 (3–7). At first sight, the expansion of NHIF coverage during that period explains the significant increase of drug spending. The provision of several categories of innovative medicinal therapies (such as rare disease and some cancer drugs) was transferred from the Ministry of Health to NHIF in 2011 and 2012, respectively. Those were all included in the outpatient drug budget category, thus increasing its spending share in absolute and relative terms. Inpatient cancer drugs were also established as a separate cost item to be funded by NHIF in 2012. This budget category alone was expected to stand at up to 100 million EUR in 2014.

While nominally not all outpatient drugs paid by NHIF are innovative, Bulgarian stakeholders have generally attributed deficit spending to outpatient medicinal therapies for rare disease and cancer. This is mostly due to the higher cost per patient and in total of those drugs. A recently published study reported important levels of cost and utilization uncertainty for some of those medicinal therapies (8). NHIF was already experiencing a budget deficit, so this insecurity of innovative therapies has contributed for the restart of the political debate in the country about NHIF expenditures.

National Health Insurance Fund deficit spending on drug therapies was expected to be around 26.6% in 2014: 488 millions EUR spent instead of 386 millions EUR initially allocated (Figure 1) (6, 7). Outpatient drug deficit spending was 34% in 2011: 268 million EUR spent against 200 million EUR allocated (3, 7). These numbers strongly alarmed Bulgarian health authorities who adopted legal amendments in drug pricing and reimbursement regulations (2). This new policy framework seemed successful at first – outpatient drug costs deficit was reduced in 2012 and 2013 (4, 5, 7). Nevertheless, outpatient drug deficit spending increased again in 2014, counting for an overspending of 76 million EUR (a deficit of 25%) (6, 7). From their inclusion in the mandatory health insurance, inpatient cancer drugs demonstrated consistent spending deficit too – 102% (a deficit spending of 30 million EUR) in 2012, 80% (a deficit spending of 37 million EUR) in 2013, and 35% (a deficit spending of 26 million EUR) in 2014 (3–6). These fluctuations can not be linked to changes in inflation (a relatively short period of 4 years) and/or currency exchange rate (Bulgaria has a currency board that maintains a fixed exchange rate with the euro).

## Rationale of Performance-Based Reimbursement

In a context of fiscal austerity, timely access to innovative therapies has to be balanced against the priorities and resources of the health system. Epidemiological, economical, and clinical uncertainty of innovative health technologies imposes deeper reflections in the process of public health priority setting and resource allocation (9, 10). When regulating access, health authorities and payers demand evidence on the number of patients to be treated, costs, and health gains (11). For these reasons, different decision support tools are explored to maximize the health benefits of the costs incurred, while mitigating the risk of overspending.

Risk-sharing agreements (RSA) are performance-based reimbursement schemes, in which the price, level, or nature of reimbursement are tied to future performance measures of clinical or intermediate endpoints ultimately related to patient quality or quantity of life (12–15). Generation and collection of new evidence is a key component of these contracts between manufactures and payers. Real-world data on the therapy’s performance subsequently assist in making informed decisions on access and coverage.

Classification of RSA relies on the type of results to be achieved: these agreements could be either health or non-health outcome-based (12). Health outcome-based RSA are linked to the achievement and/or proof of certain health benefits in a patient population for a period of time, whereas non-health outcome-based RSA are mostly tied to negotiating price and/or consumption levels. Non-health outcomes-based RSA are, however, mainly guided by financial considerations, without taking into account the health benefits at individual and population level. This practice itself does not contribute for improving the national health system’s effectiveness, as well as no new evidence is generated.

Health outcomes-based RSA are often split into two main categories (12, 13): conditional coverage, where coverage is granted conditional on the initiation of a program of data collection, and performance-linked reimbursement, where reimbursement level for covered products is tied to the measure of real-world clinical outcomes. Under a conditional coverage scheme, reimbursement decision is conditioned upon the collection of additional population level evidence, from a pre-specified scientific study, to support continued, expanded, or withdrawal of coverage. The second category, performance-linked reimbursement, is characterized by outcomes guarantees, in which the manufacturer provides rebates, refunds, or price adjustments if the product fails to meet the agreed upon outcome targets. In practice, however, RSA very often include components from both subcategories depending on the uncertainty that is being addressed.

## Prospects of Risk-Sharing Agreements for Innovative Therapies in a Context of Deficit Spending in Bulgaria

Improving patients’ access to new therapies is legitimate and in line with progress and innovation in medicine. Despite well documented trend to improve Bulgarian patients’ access to innovative therapies, availability and accessibility of those therapies largely remain limited compared to other EU member states (8). Of course, Bulgarian national health system has to operate within largely fewer and very limited resources. Thus, any source of uncertainty that leads to significant overspending could jeopardize the overall sustainability of the local healthcare model. However, nowadays dominating concepts of health technology assessment (HTA) and evidence-based medicine focus not on patient access restrictions and cost containment, but on health outcomes surveillance and real-world evidence collection (10). As coverage policy in Bulgaria remains subject to cost-minimization, a growing number of countries switch to cost-effectiveness – to spend the available resources wisely, in a way that will generate the greatest amount of health benefits to the greatest number of people (16–18). Reference pricing, centralized tenders and budgetary constraints do lower drug expenditure indeed, but eventually they all lack the effective control of outcomes obtained. In fact, these policy tools offer a temporary solution, which ultimately does not eliminate the risk of budget deficit, as observed in Bulgaria.

When rare diseases and cancer medicinal therapies were transferred to NHIF, the payer took measures to control uncertainty in cost and utilization. NHIF determined clinical criteria to be met in order to initiate and then to continue a therapy. Coverage is renewed every 6 months upon achieving predefined clinical outcomes. This mechanism falls in the category of conditional treatment continuation, which is a standard feature in many RSA. Such scheme ensures that only patients that benefit from treatment remain on treatment (12). However, a serious shortage of the approach, currently applied by NHIF, is the lack of management of the potential overspending that may occur. Linking reimbursement status to performance does not directly address this issue. Another drawback of the present practice in Bulgaria is the evidence gap about innovative therapies. NHIF is monitoring, in fact, a basic set of surrogate outcomes in patients in order to continue reimbursement, but no efforts are made to assess and appraise these real-world data in aggregate and to use this new knowledge in policy-making. In this context, evidence collection is a prerequisite for overcoming difficulties in transparency, legitimacy, and feasibility of priority setting and resource allocation in the field of public health.

Risk-sharing agreements are conceived as a response to all the above mentioned concerns (19). We call for the legal definition and practical implementation of RSA in Bulgaria. This mechanism is essential for the sustainable access to innovative medicinal therapies in the country. A hypothetical framework of RSA should include the following elements: early dialog and fast-track first-stage evaluation, post-marketing monitoring and patient registry, independent HTA report, and final informed reimbursement decision-making. The application of RSA should begin with an initial, more implicit evaluation of innovative health technologies. The main reason is the fact that most of these therapies are often the very first therapeutic option available for the patients in question (20). RSA with mandatory post-marketing surveillance will allow patients to start therapy early, thereby avoiding clinical complications and further medical expenses. At the same time, regulators and payers can get data on the real-world effectiveness and utilization of the product (21). Last but not least, RSA should contain an agreed mechanism for reducing the risk of overspending, sharing the burden of budget deficit among payers and manufacturers.

Lack of reliable epidemiological, clinical, and economic evidence, generated in local settings, is a substantial obstacle for effective planning and management of healthcare costs in Bulgaria. Especially in the case of rare diseases, the small number of patients and the impracticability to conduct large-scale randomized controlled clinical trials call for alternative study designs to generate and collect new evidence. RSA experience demonstrates that post-marketing studies and patient registries are the most appropriate tool for streamlining the processes of HTA and reimbursement decision-making for innovative therapies. Evidence generated from such studies at national level is more consistent and reliable, because it reflects the specifics of the local population and health system (9, 10). Bulgarian health authorities should actively promote the collection of real-world evidence, which is fundamental for rigorous and objective HTA, as well as for subsequent final reimbursement-decision-making.

## Conclusion

An access scheme that combines features from both health and non-health outcome-based RSA achieves two objectives simultaneously. First, it would effectively restrict the possibility of budget deficit in the healthcare system. Second and even more importantly, it would allow coverage decisions to be consistent and coherent, following a transparent procedure and clear criteria. Generation and collection of new epidemiological, clinical, and economic real-world evidence ensure that inform reimbursement decisions have been made and costs incurred have produced greatest benefits to a greatest number of people.

## Author Contributions

Both authors contributed to the publication of this opinion paper.

## Conflict of Interest Statement

The authors declare that the research was conducted in the absence of any commercial or financial relationships that could be construed as a potential conflict of interest.
